# The Röntgen Society

**Published:** 1900-11-10

**Authors:** 


### THE RÖNTGEN SOCIETY.

At a meeting of the Röntgen Society, held at 20 Hanover Square last Friday, Dr. Macintyre, of Glasgow, delivered his presidential address. Speaking of the statements which have lately been made in various quarters concerning the limited value of the X-rays in medical practice, he said that considering the work which had been done in fractures, dislocations, exploration of cavities, diseases of bone, investigations of the thoracic region in regard to aneurism, changes in the lungs, diseased glands, &c., he thought it was difficult to see cause for dissatisfaction unless it were that too high an estimate had been made at the beginning of what was a very recent but happily a very progressive science. He added that the X-rays had been found very useful in the military hospitals at the base and on the lines of communication, although not actually on the field, and that there was now a regulation list of apparatus for army work, of which seventeen sets had been sent ont to South Africa.

